# 3-*tert*-Butyl-5,6,8-trinitro­naphtho[1,8a,8-*cd*][1,2]dithiole

**DOI:** 10.1107/S160053680800946X

**Published:** 2008-04-10

**Authors:** Yuqin Jiang, Xiangjian Wan, Yongsheng Chen

**Affiliations:** a Key Laboratory for Functional Polymer Materials and Center for Nanoscale Science and Technology, Institute of Polymer Chemistry, College of Chemistry, Nankai University, Weijin Road No. 94, Tianjin, People’s Republic of China

## Abstract

Nitration of 2,7-di-*tert*-butyl­naphthalene 1,8-disulfide with fuming nitric acid in 1:3 molar ratio gives the title compound, C_14_H_11_N_3_O_6_S_2_. A tape motif is formed by weak head-to-tail inter­actions (3.131 Å) between S and NO_2_ O atoms of a symmetry-related mol­ecule.

## Related literature

For related literature, see: Barltrop *et al.* (1954 ▶); Claeson *et al.* (1960 ▶); Shigeru *et al.* (1982 ▶); Smiles & Price (1928 ▶); Stepanov *et al.* (1977 ▶); Tesmer & Vahrenkamp (2001 ▶); Zweig & Hoffman (1965 ▶); Ashe *et al.* (1994 ▶).
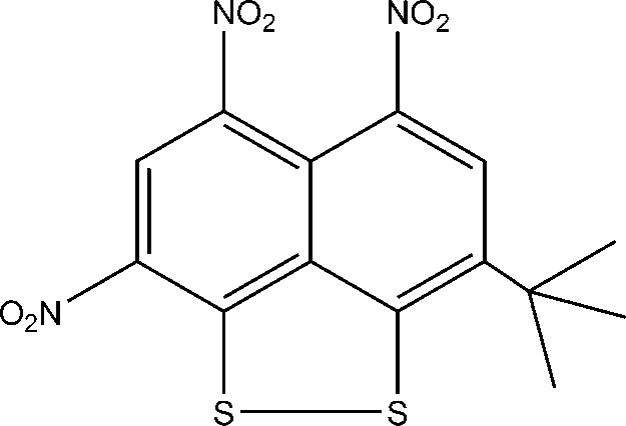


## Experimental

### 

#### Crystal data

C_14_H_11_N_3_O_6_S_2_
*M* *_r_* = 381.38
Monoclinic, 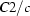

*a* = 19.477 (3) Å
*b* = 20.754 (4) Å
*c* = 8.1658 (14) Å
β = 105.909 (3)°
*V* = 3174.4 (10) Å^3^
*Z* = 8
Mo *K*α radiation
μ = 0.37 mm^−1^
*T* = 294 (2) K
0.26 × 0.24 × 0.10 mm

#### Data collection

Bruker SMART CCD area-detector diffractometer
Absorption correction: multi-scan (*SADABS*; Bruker, 1997 ▶) *T*_min_ = 0.876, *T*_max_ = 0.964
8118 measured reflections
2803 independent reflections
1856 reflections with *I* > 2σ(*I*)
*R*_int_ = 0.037

#### Refinement

*R*[*F* ^2^ > 2σ(*F* ^2^)] = 0.038
*wR*(*F* ^2^) = 0.108
*S* = 1.03
2803 reflections
229 parameters
H-atom parameters constrained
Δρ_max_ = 0.23 e Å^−3^
Δρ_min_ = −0.26 e Å^−3^

### 

Data collection: *SMART* (Bruker, 1997 ▶); cell refinement: *SAINT* (Bruker, 1997 ▶); data reduction: *SAINT*; program(s) used to solve structure: *SHELXS97* (Sheldrick, 2008 ▶); program(s) used to refine structure: *SHELXL97* (Sheldrick, 2008 ▶); molecular graphics: *SHELXTL* (Sheldrick, 2008 ▶); software used to prepare material for publication: *SHELXTL*.

## Supplementary Material

Crystal structure: contains datablocks global, I. DOI: 10.1107/S160053680800946X/pk2089sup1.cif

Structure factors: contains datablocks I. DOI: 10.1107/S160053680800946X/pk2089Isup2.hkl

Additional supplementary materials:  crystallographic information; 3D view; checkCIF report

## Figures and Tables

**Fig. 1.**
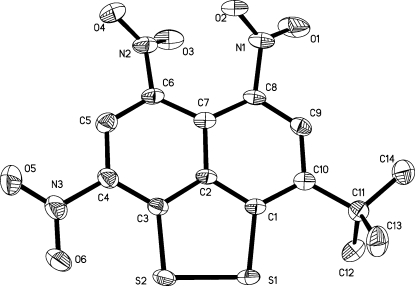
The structure of the title compound showing 30% probability ellipsoids.

**Fig. 2.**
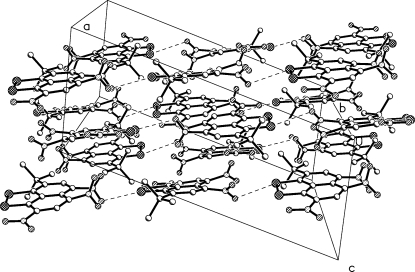
Packing structure of the title compound.

## supplementary crystallographic information

### Comment

Naphthalene-1,8-disulfide is a bright-red crystalline compound (Smiles *et al.*, 1928; Zweig *et al.*, 1965) due to the introduction of S—S group into the naphthalene ring leading to a considerable bathochromic shift of the absorption bands characteristic of disulfides in the electronic spectrum (Barltrop *et al.*, 1954; Claeson *et al.*, 1960). There are only a few reports concerning the nitration of naphthalene-1,8-disulfide (Stepanov *et al.*, 1977; Shigeru *et al.*, 1982). In an attempt to nitrate 2,7-di-*tert*-butyl-naphthalene-1,8-disulfide(BNT) (Tesmer *et al.*, 2001) with fuming nitric acid in an attempted synthesis of 3,8-di-*tert*-butyl-5,6-dinitro-naphtho[1,8-cd][1,2]dithiole, the title compound was obtained unexpectedly. Herein, we present its preparation and single-crystal structure (Fig. 1).

The crystal was obtained by recrystallization from ethyl acetate.  The length of the S—S bond is 2.0627 (10) Å, which is consistent with that of analogues reported  by Arthur *et al.* (1994).  The three rings form a slightly non-perfect plane owing to the asymmetric substitution of nitro and *tert*-butyl groups. The dihedral angles between the rings are: A/B, 3.9 (3) °; A/C, 2.3 (3) °; B/C, 3.0 (3) °. A perspective view of the packing is shown in Figure 2. A slightly wavy tape motif is formed by the head-to-tail weak interactions between S1 and O2 of a molecule related via  0.5+x,0.5-y,0.5+z.

### Experimental

To a solution of BNT (3.02 g, 10 mmol) in acetic acid (50 mL), fuming nitric acid (30 mmol) was added. The reaction mixture was stirred for 0.5 h and cooled to room temperature. The precipitate was collected by filtration and recrystallized from ethyl acetate to give the title compound as red crystals, yield 80%.

### Refinement

H atoms were found in difference Fourier maps and subsequently placed in idealized positions with constrained C-H distances of 0.96 Å (RCH_3_) and 0.93 Å (C_Ar_H) with U_iso_(H) values set to either 1.5U_eq_ (RCH_3_) or 1.2U_eq_ of the attached C atom.

### Crystal data

**Table:**

C_14_H_11_N_3_O_6_S_2_	*F*_000_ = 1568
*M**_r_* = 381.38	*D*_x_ = 1.596 Mg m^−^^3^
Monoclinic, *C*2/*c*	Mo *K*α radiation λ = 0.71073 Å
Hall symbol: -C 2yc	Cell parameters from 2099 reflections
*a* = 19.477 (3) Å	θ = 2.7–25.8º
*b* = 20.754 (4) Å	µ = 0.37 mm^−^^1^
*c* = 8.1658 (14) Å	*T* = 294 (2) K
β = 105.909 (3)º	Block, red
*V* = 3174.4 (10) Å^3^	0.26 × 0.24 × 0.10 mm
*Z* = 8	

### Data collection

**Table:**

Bruker SMART CCD area-detector diffractometer	2803 independent reflections
Radiation source: fine-focus sealed tube	1856 reflections with *I* > 2σ(*I*)
Monochromator: graphite	*R*_int_ = 0.037
*T* = 294(2) K	θ_max_ = 25.0º
φ and ω scans	θ_min_ = 1.5º
Absorption correction: multi-scan(SADABS; Bruker, 1997)	*h* = −19→23
*T*_min_ = 0.876, *T*_max_ = 0.964	*k* = −24→22
8118 measured reflections	*l* = −9→9

### Refinement

**Table:**

Refinement on *F*^2^	Secondary atom site location: difference Fourier map
Least-squares matrix: full	Hydrogen site location: inferred from neighbouring sites
*R*[*F*^2^ > 2σ(*F*^2^)] = 0.038	H-atom parameters constrained
*wR*(*F*^2^) = 0.108	*w* = 1/[σ^2^(*F*_o_^2^) + (0.0578*P*)^2^] where *P* = (*F*_o_^2^ + 2*F*_c_^2^)/3
*S* = 1.03	(Δ/σ)_max_ = 0.001
2803 reflections	Δρ_max_ = 0.24 e Å^−^^3^
229 parameters	Δρ_min_ = −0.25 e Å^−^^3^
Primary atom site location: structure-invariant direct methods	Extinction correction: none

### Special details

**Table:**

Geometry. All e.s.d.'s (except the e.s.d. in the dihedral angle between two l.s. planes) are estimated using the full covariance matrix. The cell e.s.d.'s are taken into account individually in the estimation of e.s.d.'s in distances, angles and torsion angles; correlations between e.s.d.'s in cell parameters are only used when they are defined by crystal symmetry. An approximate (isotropic) treatment of cell e.s.d.'s is used for estimating e.s.d.'s involving l.s. planes.
Refinement. Refinement of *F*^2^ against ALL reflections. The weighted *R*-factor *wR* and goodness of fit *S* are based on *F*^2^, conventional *R*-factors *R* are based on *F*, with *F* set to zero for negative *F*^2^. The threshold expression of *F*^2^ > σ(*F*^2^) is used only for calculating *R*-factors(gt) *etc*. and is not relevant to the choice of reflections for refinement. *R*-factors based on *F*^2^ are statistically about twice as large as those based on *F*, and *R*- factors based on ALL data will be even larger.

### Fractional atomic coordinates and isotropic or equivalent isotropic displacement parameters (Å^2^)

**Table:**

	*x*	*y*	*z*	*U*_iso_*/*U*_eq_	
S1	0.22607 (3)	0.26843 (3)	0.74057 (9)	0.0476 (2)	
S2	0.21867 (3)	0.17028 (3)	0.77160 (9)	0.0490 (2)	
O1	−0.10572 (11)	0.34597 (11)	0.3300 (3)	0.0838 (7)	
O2	−0.12517 (9)	0.26776 (9)	0.4864 (2)	0.0525 (5)	
O3	−0.10009 (10)	0.18790 (11)	0.2446 (2)	0.0661 (6)	
O4	−0.12944 (10)	0.12220 (10)	0.4212 (3)	0.0665 (6)	
O5	0.08457 (13)	0.00261 (10)	0.7190 (3)	0.1019 (9)	
O6	0.18753 (12)	0.05183 (10)	0.7857 (3)	0.0809 (7)	
N1	−0.08510 (11)	0.30111 (12)	0.4295 (3)	0.0494 (6)	
N2	−0.08763 (12)	0.15858 (12)	0.3800 (3)	0.0513 (6)	
N3	0.12234 (14)	0.05040 (12)	0.7216 (3)	0.0693 (8)	
C1	0.13641 (12)	0.28171 (11)	0.6422 (3)	0.0349 (6)	
C2	0.09381 (12)	0.22503 (11)	0.6122 (3)	0.0332 (6)	
C3	0.12897 (13)	0.16621 (11)	0.6715 (3)	0.0378 (6)	
C4	0.08893 (14)	0.10991 (12)	0.6509 (3)	0.0469 (7)	
C5	0.01708 (14)	0.10991 (13)	0.5605 (3)	0.0487 (7)	
H5	−0.0084	0.0714	0.5431	0.058*	
C6	−0.01603 (12)	0.16565 (12)	0.4978 (3)	0.0402 (6)	
C7	0.01938 (12)	0.22687 (11)	0.5293 (3)	0.0349 (6)	
C8	−0.00856 (12)	0.28886 (12)	0.4886 (3)	0.0378 (6)	
C9	0.03362 (13)	0.34351 (12)	0.5203 (3)	0.0418 (6)	
H9	0.0116	0.3831	0.4882	0.050*	
C10	0.10644 (13)	0.34324 (12)	0.5964 (3)	0.0388 (6)	
C11	0.14946 (14)	0.40632 (12)	0.6359 (3)	0.0445 (7)	
C12	0.20882 (15)	0.40735 (14)	0.5453 (4)	0.0603 (8)	
H12A	0.2363	0.4462	0.5746	0.090*	
H12B	0.1880	0.4058	0.4244	0.090*	
H12C	0.2394	0.3707	0.5803	0.090*	
C13	0.18252 (17)	0.41303 (14)	0.8288 (3)	0.0655 (9)	
H13A	0.2145	0.3777	0.8691	0.098*	
H13B	0.1453	0.4127	0.8853	0.098*	
H13C	0.2084	0.4529	0.8523	0.098*	
C14	0.10141 (16)	0.46540 (13)	0.5768 (4)	0.0678 (9)	
H14A	0.1295	0.5040	0.6038	0.102*	
H14B	0.0643	0.4661	0.6338	0.102*	
H14C	0.0804	0.4631	0.4560	0.102*	

### Atomic displacement parameters (Å^2^)

**Table:**

	*U*^11^	*U*^22^	*U*^33^	*U*^12^	*U*^13^	*U*^23^
S1	0.0239 (3)	0.0561 (5)	0.0571 (5)	0.0011 (3)	0.0013 (3)	−0.0029 (3)
S2	0.0287 (4)	0.0533 (4)	0.0576 (5)	0.0105 (3)	−0.0008 (3)	−0.0063 (3)
O1	0.0463 (13)	0.1089 (19)	0.0858 (15)	0.0205 (12)	0.0004 (11)	0.0525 (15)
O2	0.0270 (10)	0.0763 (14)	0.0557 (12)	0.0019 (9)	0.0140 (9)	0.0021 (10)
O3	0.0457 (12)	0.1145 (17)	0.0317 (10)	0.0013 (11)	0.0000 (9)	−0.0013 (11)
O4	0.0399 (12)	0.0745 (15)	0.0789 (14)	−0.0160 (11)	0.0057 (11)	−0.0059 (11)
O5	0.0882 (18)	0.0412 (13)	0.155 (3)	−0.0054 (12)	−0.0030 (17)	−0.0026 (14)
O6	0.0535 (14)	0.0582 (13)	0.1134 (19)	0.0205 (11)	−0.0069 (13)	−0.0051 (12)
N1	0.0285 (12)	0.0749 (17)	0.0422 (13)	0.0080 (11)	0.0051 (10)	0.0072 (12)
N2	0.0314 (13)	0.0721 (17)	0.0468 (14)	−0.0024 (12)	0.0050 (11)	−0.0145 (12)
N3	0.0602 (18)	0.0427 (15)	0.093 (2)	0.0105 (14)	0.0005 (16)	−0.0149 (14)
C1	0.0269 (13)	0.0478 (15)	0.0289 (13)	0.0022 (11)	0.0060 (10)	−0.0008 (11)
C2	0.0247 (13)	0.0481 (15)	0.0252 (12)	0.0044 (11)	0.0042 (10)	−0.0027 (10)
C3	0.0315 (13)	0.0438 (15)	0.0352 (13)	0.0043 (11)	0.0041 (11)	−0.0106 (11)
C4	0.0418 (16)	0.0425 (16)	0.0504 (16)	0.0070 (13)	0.0027 (13)	−0.0099 (13)
C5	0.0423 (16)	0.0485 (17)	0.0507 (17)	−0.0061 (13)	0.0053 (13)	−0.0127 (13)
C6	0.0269 (13)	0.0571 (17)	0.0336 (14)	−0.0022 (12)	0.0032 (11)	−0.0081 (12)
C7	0.0292 (13)	0.0541 (16)	0.0219 (12)	0.0015 (11)	0.0079 (10)	−0.0016 (11)
C8	0.0248 (13)	0.0569 (17)	0.0303 (13)	0.0072 (12)	0.0051 (10)	0.0064 (12)
C9	0.0395 (15)	0.0478 (17)	0.0383 (14)	0.0105 (12)	0.0107 (12)	0.0116 (12)
C10	0.0350 (14)	0.0505 (17)	0.0313 (13)	0.0015 (12)	0.0097 (11)	0.0039 (11)
C11	0.0460 (17)	0.0451 (16)	0.0410 (15)	−0.0004 (12)	0.0097 (13)	0.0026 (12)
C12	0.0556 (19)	0.066 (2)	0.0591 (19)	−0.0194 (15)	0.0155 (15)	−0.0042 (14)
C13	0.094 (3)	0.0513 (18)	0.0480 (18)	−0.0007 (16)	0.0145 (17)	−0.0109 (14)
C14	0.068 (2)	0.0496 (18)	0.082 (2)	0.0001 (15)	0.0139 (17)	0.0122 (15)

### Geometric parameters (Å, °)

**Table:**

S1—C1	1.733 (2)	C5—H5	0.9300
S1—S2	2.0627 (10)	C6—C7	1.435 (3)
S2—C3	1.716 (2)	C7—C8	1.401 (3)
O1—N1	1.229 (3)	C8—C9	1.383 (3)
O2—N1	1.226 (3)	C9—C10	1.384 (3)
O3—N2	1.227 (3)	C9—H9	0.9300
O4—N2	1.223 (3)	C10—C11	1.540 (3)
O5—N3	1.231 (3)	C11—C12	1.534 (4)
O6—N3	1.234 (3)	C11—C13	1.536 (3)
N1—C8	1.458 (3)	C11—C14	1.538 (4)
N2—C6	1.468 (3)	C12—H12A	0.9600
N3—C4	1.441 (3)	C12—H12B	0.9600
C1—C10	1.411 (3)	C12—H12C	0.9600
C1—C2	1.421 (3)	C13—H13A	0.9600
C2—C3	1.419 (3)	C13—H13B	0.9600
C2—C7	1.422 (3)	C13—H13C	0.9600
C3—C4	1.389 (3)	C14—H14A	0.9600
C4—C5	1.392 (3)	C14—H14B	0.9600
C5—C6	1.355 (3)	C14—H14C	0.9600
			
C1—S1—S2	96.81 (8)	C9—C8—N1	114.8 (2)
C3—S2—S1	94.96 (9)	C7—C8—N1	122.3 (2)
O2—N1—O1	123.5 (2)	C8—C9—C10	124.3 (2)
O2—N1—C8	118.4 (2)	C8—C9—H9	117.8
O1—N1—C8	118.1 (2)	C10—C9—H9	117.8
O4—N2—O3	124.9 (2)	C9—C10—C1	114.9 (2)
O4—N2—C6	117.8 (2)	C9—C10—C11	121.5 (2)
O3—N2—C6	117.3 (2)	C1—C10—C11	123.5 (2)
O5—N3—O6	124.5 (3)	C12—C11—C13	109.5 (2)
O5—N3—C4	118.8 (3)	C12—C11—C14	108.2 (2)
O6—N3—C4	116.6 (3)	C13—C11—C14	107.3 (2)
C10—C1—C2	121.6 (2)	C12—C11—C10	110.5 (2)
C10—C1—S1	123.91 (18)	C13—C11—C10	110.1 (2)
C2—C1—S1	114.50 (17)	C14—C11—C10	111.3 (2)
C3—C2—C1	116.7 (2)	C11—C12—H12A	109.5
C3—C2—C7	121.3 (2)	C11—C12—H12B	109.5
C1—C2—C7	122.0 (2)	H12A—C12—H12B	109.5
C4—C3—C2	118.7 (2)	C11—C12—H12C	109.5
C4—C3—S2	124.31 (19)	H12A—C12—H12C	109.5
C2—C3—S2	116.97 (18)	H12B—C12—H12C	109.5
C3—C4—C5	120.9 (2)	C11—C13—H13A	109.5
C3—C4—N3	119.7 (2)	C11—C13—H13B	109.5
C5—C4—N3	119.4 (2)	H13A—C13—H13B	109.5
C6—C5—C4	120.4 (2)	C11—C13—H13C	109.5
C6—C5—H5	119.8	H13A—C13—H13C	109.5
C4—C5—H5	119.8	H13B—C13—H13C	109.5
C5—C6—C7	122.2 (2)	C11—C14—H14A	109.5
C5—C6—N2	115.5 (2)	C11—C14—H14B	109.5
C7—C6—N2	122.0 (2)	H14A—C14—H14B	109.5
C8—C7—C2	114.6 (2)	C11—C14—H14C	109.5
C8—C7—C6	129.4 (2)	H14A—C14—H14C	109.5
C2—C7—C6	116.0 (2)	H14B—C14—H14C	109.5
C9—C8—C7	122.5 (2)		
			
C1—S1—S2—C3	2.27 (11)	C1—C2—C7—C8	−3.2 (3)
S2—S1—C1—C10	176.23 (18)	C3—C2—C7—C6	−4.8 (3)
S2—S1—C1—C2	−2.85 (17)	C1—C2—C7—C6	176.2 (2)
C10—C1—C2—C3	−176.8 (2)	C5—C6—C7—C8	−173.2 (2)
S1—C1—C2—C3	2.3 (3)	N2—C6—C7—C8	13.8 (4)
C10—C1—C2—C7	2.3 (3)	C5—C6—C7—C2	7.6 (3)
S1—C1—C2—C7	−178.63 (16)	N2—C6—C7—C2	−165.4 (2)
C1—C2—C3—C4	177.5 (2)	C2—C7—C8—C9	2.6 (3)
C7—C2—C3—C4	−1.5 (3)	C6—C7—C8—C9	−176.7 (2)
C1—C2—C3—S2	−0.2 (3)	C2—C7—C8—N1	−169.64 (19)
C7—C2—C3—S2	−179.27 (17)	C6—C7—C8—N1	11.1 (4)
S1—S2—C3—C4	−179.1 (2)	O2—N1—C8—C9	−138.7 (2)
S1—S2—C3—C2	−1.51 (19)	O1—N1—C8—C9	38.6 (3)
C2—C3—C4—C5	5.7 (4)	O2—N1—C8—C7	34.1 (3)
S2—C3—C4—C5	−176.7 (2)	O1—N1—C8—C7	−148.6 (3)
C2—C3—C4—N3	−175.3 (2)	C7—C8—C9—C10	−1.0 (4)
S2—C3—C4—N3	2.3 (4)	N1—C8—C9—C10	171.8 (2)
O5—N3—C4—C3	171.9 (3)	C8—C9—C10—C1	−0.2 (4)
O6—N3—C4—C3	−6.0 (4)	C8—C9—C10—C11	−176.9 (2)
O5—N3—C4—C5	−9.0 (4)	C2—C1—C10—C9	−0.5 (3)
O6—N3—C4—C5	173.0 (3)	S1—C1—C10—C9	−179.49 (17)
C3—C4—C5—C6	−3.2 (4)	C2—C1—C10—C11	176.2 (2)
N3—C4—C5—C6	177.8 (2)	S1—C1—C10—C11	−2.8 (3)
C4—C5—C6—C7	−3.7 (4)	C9—C10—C11—C12	−121.4 (3)
C4—C5—C6—N2	169.7 (2)	C1—C10—C11—C12	62.2 (3)
O4—N2—C6—C5	46.7 (3)	C9—C10—C11—C13	117.6 (3)
O3—N2—C6—C5	−130.7 (3)	C1—C10—C11—C13	−58.8 (3)
O4—N2—C6—C7	−139.9 (2)	C9—C10—C11—C14	−1.2 (3)
O3—N2—C6—C7	42.7 (3)	C1—C10—C11—C14	−177.6 (2)
C3—C2—C7—C8	175.8 (2)		
